# Sequence identification and phylogenetic analysis of the mitochondrial genome of *Microphysogobio kiatingensis* (Cypriniformes: Cyprinidae)

**DOI:** 10.1080/23802359.2018.1456981

**Published:** 2018-04-01

**Authors:** Yuanchao Zou, Junying Zhang, Tao Zhang, Meng Xie, Tian Wu

**Affiliations:** College of Life Sciences, Neijiang Normal University, Conservation and Utilization of Fishes Resources in the Upper Reaches of the Yangtze River Key Laboratory of Sichuan Province, Neijiang, China

**Keywords:** *Microphysogobio kiatingensis*, mitochondrial genome, phylogenetic analysis

## Abstract

*Microphysogobio kiatingensis* is endemic freshwater fish of China and mainly distributed in the middle and upper reaches of the Yangtze River. The complete mitogenome of *M. kiatingensis* was first determined by using the next-generation sequencing in present study. The circular mitogenome followed the expected pattern for vertebrates, being 16,603 bp in length. It contained 13 protein-coding genes, two ribosomal RNA genes, 22 transfer RNA genes, one displacement loop (D-loop) locus and an origin of replication on the light-strand (OL). Most of the genes are encoded on the heavy strand except ND6 and eight tRNA genes. The overall base composition was 30.78% A, 26.09% T, 26.59% C, 16.54% G, with 56.87% AT. Thirteen intergenic spacers and nine gene overlaps exist in the complete mitochondrial genome. Phylogenetic analysis based on the tandem 13 coding protein genes nucleotide sequences with two different methods (maximum likelihood and neighbour-joining analysis) indicated that *M. kiatingensis* was grouped in one clade with the same genus. The complete mitogenome of *M. kiatingensis* can provide a useful data for the further research in Gobioninae. Meanwhile, it also provides help for biological genetics and classification of species.

*Microphysogobio kiatingensis* (Cypriniformes, Cyprinidae, Gobioninae, *Microphysogobio*), originated in the Leshan, Sichuan, is endemic freshwater fish of China and mainly distributed in the middle and upper reaches of the Yangtze River (Lin et al. 2018). Although *M. kiatingensis* has certain economic value, its natural population is weak (Zhou and Zhang 2006). What is worse, there is still no information reported about complete mitogenome of *M. kiatingensis*.

The complete mitochondrial DNA sequence of *M. kiatingensis* was first determined by the next generation sequencing (NGS) in this study. The specimens were collected from Chengdu, Sichuan province of China (30°55′6.08″N, 104°22′49.21″E) in September 2017 and were stored in Zoological Specimen Museum of Neijiang Normal University (accession number: 20170925BB05). A 30–40 mg fin clip was collected and preserved in 95% ethanol at 4 °C. Total genomic DNA was extracted from these caudal fins by a Tissue DNA Kit (OMEGA E.Z.N.A., Norcross, GA) following the manufacturer’s protocol. Subsequently, the genomic DNA was sequenced using the NGS, and then the mitogenome was assembled using *Abbottina rivularis* as reference.

The circular mitogenome followed the expected pattern for vertebrates, being 16,603 bp in length (GenBank Accession number MG797640). It contained 13 protein-coding genes, two ribosomal RNA genes, 22 transfer RNA genes, one displacement loop (D-loop) locus and an origin of replication on the light-strand (OL). In these genes, ND6 and eight tRNA genes were encoded on light strand while the rest of genes were encoded on heavy strand and the arrangement of these genes were the same as that found in teleosts (Hwang et al. 2014; Cheng et al. 2015). The overall base composition was 30.78% A, 26.09% T, 26.59% C, and 16.54% G, with a slight AT bias (56.87%). For 13 PCGs, ranging from 165 bp (ATP8) to 1836 bp (ND5) in length, ATG is the common initiation codon while COI began with GTG. Correspondingly, 11 PCGs stopped with the complete termination codon TAG (ND1, ND4, ND6) or TAA (ND2, COI, COII, ATP8, ATP6, ND3, ND4L, ND5), while the rest of PCGs ended with an incomplete termination codon T (COIII, Cyt b). There were 13 intergenic spacers (total 63 bp) varying from 1 to 31 bp in length and nine gene overlaps (total 25 bp) in the mitochondrial genome. Furthermore, the 22 tRNA genes were interspersed among the rRNA genes and PCGs, ranged in size from 62 bp (tRNA^Lys^) to 76 bp (tRNA^Leu^). Moreover, 12S rRNA (958 bp) and 16S rRNA (1691 bp) were located between tRNA^Phe^ and tRNA^Leu^ and separated by the tRNA^Val^. Additionally, two non-coding regions were D-loop (927 bp) and OL (31 bp), which may control mitogenome’s replication and transcription.

So far, the mitochondrial PCGs have been widely used for inferring phylogenetic relationships (Boore et al. 2005). To explore their phylogenetic relationship of *M. kiatingensis* and other Gobioninae subfamily fishes, the phylogenetic tree was constructed based on neighbour-joining (NJ) and maximum likelihood (ML) methods using the 13 PCGs (Zou et al. 2017). In addition, *Channa argus* (Channoidei, Channidae) was defined as an outgroup. The 13 taxa all belong to Cyprinidae, Gobioninae except *C. argus*. The other 12 species from three genus (*Microphysogobio*, *Abbottina*, *Sarcocheilichthys*) were divided into three clades (Figure 1). The same genus was clustered in the same branch with high support. Additionally, results suggested that *Microphysogobio* has a closer relationship with *Abbottina* than *Sarcocheilichthys*. These results may provide an important information for morphological classification of Gobioninae species and the study of biological origin.

**Figure 1.**
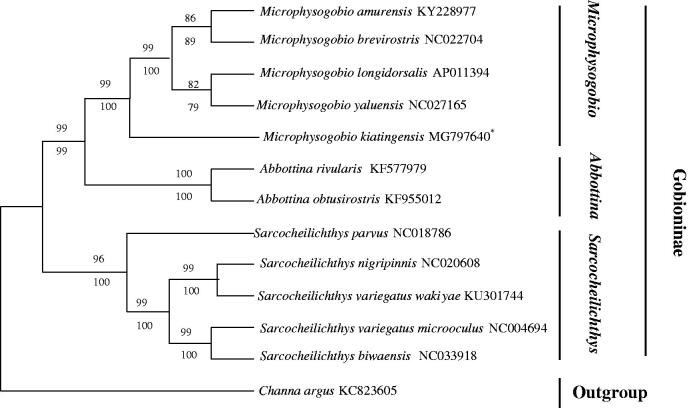
The phylogenetic analysis investigated using neighbour-joining (NJ) and maximum likelihood (ML) analyses indicated evolutionary relationships among 13 taxa based on nucleotide sequences of 13 concatenated protein-coding genes. The yielded NJ tree had a same topology as that of ML tree. *Channa argus* (GenBank: KC823605) was used as an outgroup.
